# Tumor-Educated Platelets Facilitate Thrombus Formation Through Migration

**DOI:** 10.3389/fonc.2022.857865

**Published:** 2022-02-24

**Authors:** Zheming Liu, Jing Wang, Fuben Liao, Qibin Song, Yi Yao

**Affiliations:** ^1^ Cancer Center, Renmin Hospital of Wuhan University, Wuhan, China; ^2^ Reproductive Medicine Centre, Zhongshan Hospital, Fudan University, Shanghai, China

**Keywords:** tumor-educated platelets, hypercoagulable state, cell migration, cancer-associated thrombosis, plasma

## Abstract

Platelets are small anucleate cells that circulate in the blood and form thrombi. Tumor-educated platelets are the platelets derived from cancer patients. Although many have reported that tumor-educated platelets are associated with cancer-associated thrombosis, their function in this process is poorly understood. Here we first collect the clinical data from 100 different cancer patients, showing that cancer patients are in a hypercoagulable state. Our experiment shows that tumor-educated platelets from melanoma-burdened mouse models can migrate faster and longer, forming more clots (thrombus). However, the plasma from tumor mice can inhibit platelet migration. The RNA sequence profile of tumor-educated platelets shows that many genes associated with cell migration and cell skeleton expressed significantly higher. Our research offers a new insight into the tumor-educated platelets to better understand the thrombus formation.

## Introduction

Nearly 100 billion platelets will be shed into the blood lumen per day to maintain their count and hemostasis (1). Through the binding of glycoproteins Ib and VI with collagen and von Willebrand factor (vWF), platelets will be activated to release more coagulation factors such as ADP and thromboxane A2 (TXA2) and express integrin αIIbβ3 that facilitate platelet adhesion (2–7). Platelets exert a profound effect on immunologic modulation, inflammation, anti-bacteria, and cancer (8–10). The concept of tumor-educated platelets (TEPs) is firstly used to describe the platelets in the tumor-burdened body that is correlated with thrombus formation (cancer-associated thrombosis) and direct interaction with tumor cells (11).

Platelets can conveniently transport the microvesicles and produce nearly 70% to 90% of them in the blood (12, 13). The granules in TEPs contain large amounts of tumor-derived proteins, which mediate the communication between tumors and organs (14, 15). Thomas Wurdinger reported that TEPs contain many biomarkers of six different tumors, among which many could be accurately detected (16).

As we mentioned above, CAF frequently happens in cancer patients. Compared with those who do not have cancer, cancer patients are nearly sevenfold higher at risk to form thrombus than them. Cancer patients are prone to develop thrombi due to their hypercoagulable state, which is contributed by multiple factors (14, 17). Although many studies have focused on this, the prothrombotic state’s mechanism remains to be unraveled.

Cell migration is a spatiotemporally modulated system. Florian established a sophisticated and comprehensive platelet locomotion system that illuminates the detailed process of platelet migration (9). Following protrusion extension, platelets adhere to a unique substrate when activated by ADP within the environment of cytoplasm calcium (Ca^2+^). Then, activated platelets triggered by thromboxane A2 will migrate over a fibrinogen-rich substrate.

Here we show that tumor-educated platelets isolated from the melanoma-burdened mice could migrate faster and longer and quickly form clots. The plasma that contains multiple anticoagulant factors presented a more substantial effect in balancing this pathophysiological process. mRNA transcript analysis of TEPs showed multiple upregulated genes associated with platelet activation, coagulation, cell skeleton, etc.

## Materials and Methods

### Cell Line and Cell Culture

Mouse melanoma B16-F10 cells (obtained from ATCC) were grown in Roswell Park Memorial Institute (RPMI) 1640 (Gibco, Grand Island, NY, USA, 11875093), mixed with 10% fetal bovine serum (FBS, Gibco, 26140079) and 1% penicillin/streptomycin (Sigma, San Francisco, CA, USA, P4333), in 37°C, 5% CO_2_.

### Tumor Mouse Model

The National Research Council’s Guide follows all mouse experiments for the Care and Use of Laboratory Animals. 5×10 5 B16-F10 cells were injected subcutaneously into the flank of legs of the C57BL/6 mice (10 weeks age, male). Anesthesia regimens were used as reported (18).

### Platelet and Plasma Isolation

Blood draws followed the reported protocol. The blood was mixed with an equal volume of pH 6.5 Tyrode’s buffer and centrifuged at 70g, 20 min, room temperature (RT), without brake. The 1-ml plasma-rich platelets (PRP) were separated into two parts: one was washed with 1.5 ml pH 7.4 Tyrode’s buffer and centrifuged at 1,200g for 10 min with brake, RT. The supernatant was discarded, and the pellet was resuspended in 400 μl pH 6.5 Tyrode’s buffer to obtain the TEPs. Another half of the PRP would be centrifuged directly at 1,200g for 10 min with brake, RT. The supernatant was then mouse plasma.

### Platelet Migration and Track Analysis

Glass coverslip ibidi sticky slides were prepared as reported (19). 104/μl mouse platelet together with 4 μM ADP (Aladdin) 30 μg/ml casein (Sigma), 40 μg/ml human fibrinogen (Sigma), 200 μM Cacl_2_, and 2 μM U46619 (Enzo Life Sciences, Farmingdale, NY, USA) was mounted together in a total volume of 240 μl pH 7.4 Tyrode’s buffer. We used Fiji and chemotaxis migration to analyze cell tracks (9).

### mRNA Transcript Analysis

Platelet RNA isolation followed the kit protocol of Qiagen RNeasy Micro Kit (Cat. no. 74004). Using StringTie software to predict the transcripts of all samples, and then using RSEM software to calculate the results of the comparison of bowtie2, we got the number of reads of each transcript for each sample and performed FPKM (Fragments Per Kilobase per Million bases) conversion. The analysis software is the R language package DEseq2 (20). The screening threshold is FDR (false discovery rate) <0.05, log FC (fold change (condition 2/condition 1) for a gene) >1, or log FC<-1. Hypergeometric distribution was used for gene ontology (GO) enrichment analysis, and the GO term with Q-value ≤0.05 was selected as the significantly enriched GO entry. Gene Set Enrichment Analysis was performed using the GSEA software (https://www.broadinstitute.org/gsea/) with permutation = gene set, metric = Diff_of_classes, metric = weighted, #permutation = 2500.

### Statistical Analysis

All data are analyzed by the SPSS software and are presented as means ± SEM. Student’s unpaired t-test and repeated ANOVA are used in this study as the statistical methods, and p values<0.05 are considered statistically significant.

## Results

### Cancer Patients Are With a Hypercoagulable State

The retrospective study was conducted in Zhongshan Hospital, Shanghai. Thromboelastogram (TEG) (**Supplementary Figure 1A**) and platelet aggregation assay data were collected from 100 patients each, who were diagnosed with different solid cancers during January 2021 to September 2021 (**Table 1**). Ethical approval (B2020-330R) was obtained from the Zhongshan Hospital Research Ethics Committee.

**Table 1:** Thromboela-stogram (TEG) and platelet aggregation assay.

TEP assay	Statistics		p value	Aggregation assay	Statistics		p value
	Control (n = 100)	Patients (n = 100)			Control (n = 100)	Patients (n = 100)	
Age (year)	44.93 ± 16.79	59.65 ± 12.36		Age	48.04 ± 18.89	55.61 ± 12.23	
Sex	50 male, 50 female	66 male, 34 female		Sex	58 male, 42 female	63 male, 37 female	
Thoracic cancer		59				15	
Gastrointestinal cancer		37				81	
Genitourinary cancer		2				4	
Sarcoma		2				0	
Angle	67.35 ± 2.93	70.24 ± 9.23	0.0032**	AvrAR(AA)	56.64 ± 11.32	46.14 ± 23.17	0.0014**
CI	0.25 ± 0.93	0.981 ± 2.76	0.0128*	AvrAR(ADP)	48.70 ± 11.40	36.17 ± 17.50	ns
K	1.59 ± 0.27	1.57 ± 1.29	ns	MPV(AA)	10.60 ± 0.89	11.10 ± 1.76	0.0142*
MA	59.1 ± 4.27	61.23 ± 10.00	ns	MPV(ADP)	10.62 ± 0.87	11.11 ± 1.77	0.0141*
R	5.69 ± 0.77	5.31 ± 1.05	0.0043**	MaxAR(AA)	58.80 ± 9.99	50.09 ± 21.78	<0.0001****
				MaxAR(ADP)	39.89 ± 17.14	51.16 ± 11.33	0.0463*

Data are expressed as mean ± SD, n = 100; unpaired Student t-test.

*p < 0.05; **p < 0.01; ****p < 0.0001; ns, no statistical significance.

TEG data showed that cancer patients presented coagulation with a shorter reaction time (R) and higher coagulation index (CI) relative to the control group. The α angle is also more prominent in the cancer patient group than in the control group (**Supplementary Figures 1B**–**D**), whereas K time and maximum amplitude (MA) have no noticeable difference (**Supplementary Figures 1E, F**). The platelet aggregation assay showed that cancer patients in the AA group had a higher average, maximum aggregation rate, and more considerable mean platelet volume (**Supplementary Figures 1G, H, K**). However, in the ADP group, only mean platelet volume in the cancer patient group is significantly different (**Supplementary Figures 1I, J, L**). Collectively, these data represent that cancer patients are in a hypercoagulable state.

### TEP Migration Facilitates Clot (Thrombosis) Formation

**Figure 1:**
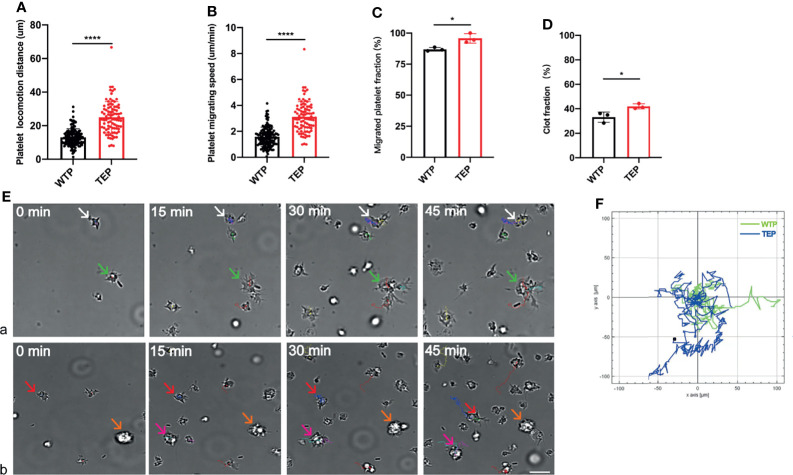
Platelet locomotion assay. **(A)** TEPs could migrate longer; **(B)** TEPs could migrate faster than the control group; **(C)** TEPs were eager to migrate; **(D)** TEPs were more efficient to form clots; **(Ea)** the WTP can migrate to contact with each other (white and green arrows); **(Eb)** TEPs can migrate to form clots (red and pink arrows), and some clots are formed before they attached to the substrate (orange arrows). Different colors are used to mark different platelet tracks. The scale bars represent 7 μm; **(F)** chemotaxis migration analysis data showed that TEPs achieved a longer track (blue) in relative to WTPs (green); n = 3 experiments; mean ± SEM; Students-t and ANOVA test; *p < 0.05; ****p < 0.0001; plt, platelet; WTP, wild-type platelet; TEP, tumor-educated platelet.

We established the platelet locomotion system based on Florian to map thrombosis formation (9, 19). We observed the whole locomotion process of mouse-derived platelets, including spreading, polarization, and migration (**Supplementary Videos 1** and **2**). Unlike wild-type platelets (WTPs), many TEPs formed clots before the locomotion started. TEPs did migrate longer and faster than WTP (**Figures 1A, B**). Moreover, TEPs were more eager to migrate and more efficiently to form clots than WTP (**Figures 1C, D**). Here we name the clot when two or more platelets contact each other, which process that *in vivo* may form a thrombus. Migrating WTPs could contact each other without fusion (**Figure 1Ea** green and white arrows and video 1). Instead, TEP could contact firmly **(Figure 1Eb** red arrows) and further fuse (**Figure 1Eb** pink arrows); this may further attract other platelets or clots to form more extensive clots (**Figure 1Eb** orange arrows, video 2). More interesting is that the big clot could still move at high speed (**Figure 1Eb** yellow and pink arrows, video 2). The chemotaxis migration tool from ibidi depicts the cell migration track (**Figure 1F**). Conclusively, the TEPs may be more easily to form and consolidate thrombosis.

### Plasma Balances the Coagulable State

The hypercoagulable state is a pathophysiological phenomenon that is affected by multiple factors. We next examined whether the change of plasma or both platelet and plasma finally leads to the hypercoagulable state (**Supplementary Video 3**). Different from their migration in Tyrode’s buffer, platelets formed cilium-like pseudopodium and moved without complete attachment to the substrate. Moreover most platelets migrated within a small range in the plasma.

**Figure 2:**
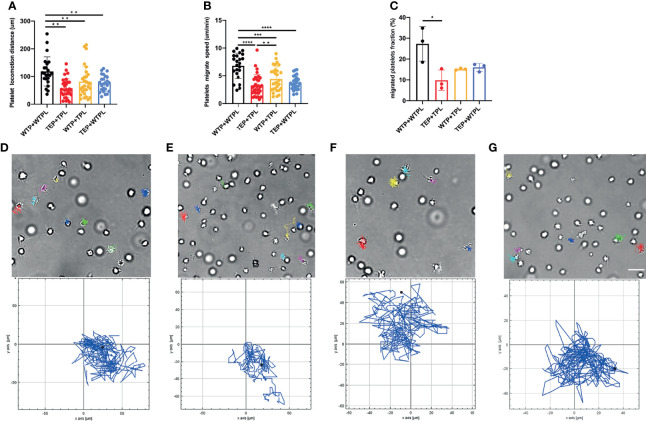
Platelet migration in plasma. **(A)** WTP+WTPL migrated the longest distance among all the four groups. **(B)** WTP+WTPL migrated fastest in the four groups; WTP+TPL could migrate faster than TEP+TPL. **(C)** There were more migrated platelets in WTP+WTPL than in TEP+TPL, migrated platelet fraction = migrated platelets/the platelets in the whole focus. **(D–G)** Platelets migrated track of WTP+WTPL **(D)**, TEP+TPL **(E)**, WTP+TPL **(F)**, and TEP+WTPL **(G)**, respectively. The cell tracks relative to each group are under each cell-migration picture. Different colors are used to mark different platelet tracks. The scale bar represents 7 μm; n = 3 experiments; mean ± SEM; Students-t and ANOVA test; *p < 0.05; **p < 0.01; ***p < 0.001; ****p < 0.0001; plt, platelet; WTP, wild-type platelet; TEP, tumor-educated platelet; WTPL, wild-type mice plasma; TPL, tumor mouse plasma.

Given the previous reports that plasma contains many coagulation factors, we estimated that the TEPs would behave more robustly when they migrate in plasma (21). However, the TEP migrating track is shorter and with a relatively slower speed (**Figures 2A, B**). More obviously, very few TEPs can migrate to form clots (**Figure 2C**) in their physical plasma environment (**Supplementary Video 4**).

To check whether the plasma interferes with the migration of TEPs, we conducted the WTP migration assay within the B16 mouse-derived plasma (TPL). The results showed that compared with the WTP+WTPL group, WTP did migrate slower and shorter in TPL (**Figures 2A, B**). Compared with the TEP+TPL group, the WTP can still migrate faster in TPL, but the migrating distance has no statistical difference. To further explain the finding that the TPL may inhibit the migration of platelets, we then performed the TEP migration within the WTPL. The results showed that only the WTP+WTPL group could move faster and longer than WTP. On both migrating distance and speed sides, the TEP+WTPL group showed no noticeable difference from the other two groups. The chemotaxis migration data presented a disorderly and unsystematically cell track (**Figures 2D**–**G** and **Supplementary Videos 3**–**6**). These data also supported our hypothesis that plasma isolated from tumor-burdened mice may contain more inhibitors to prevent platelet migration.

### TEPs Are Genetically Modulated

**Figure 3:**
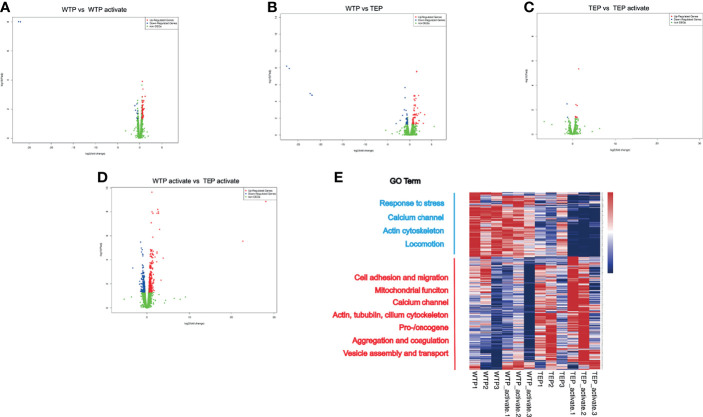
The RNA sequence profile of tumor-educated platelets. **(A)** Once WTPs were activated, there were in all 54 genes upregulated and 11 genes downregulated compared to their inactivated state. **(B)** Compared to WTP, there were 66 genes upregulated and 39 downregulated in TEP. **(C)** Only 13 genes were upregulated and two downregulated in active TEPs compared to inactivated TEPs. **(D)** Relative to the activated WTPs, there were 358 upregulated and 203 downregulated genes in the activated TEPs. **(E)** The heatmap of the RNA transcript. The genes in WTPs and activated WTPs enriched mainly in genes associated with response to stress, calcium channel, actin cytoskeleton, and locomotion. In TEPs and activated TEPs, the differential genes enriched in cell adhesion and migration, mitochondrial function, calcium channel, actin, cilium cytoskeleton, pro-/oncogene, aggregation, and coagulation, vesicle assembly, and transport. GO, gene ontology; DEG, differential expression gene; WTP, wild-type platelet; TEP, tumor-educated platelet.

We assumed that the significant difference between TEP and WTP may be due to the tumor’s education. The total mRNA from WTPs and TEPs was captured to unravel the ultimate reason. The volcano plots showed that many genes in WTPs were regulated when they were activated (**Figure 3A**), and TEPs differ significantly from WTPs in differential gene numbers (**Figure 3B**). Activated TEPs differ less from the original TEPs in the regulated gene numbers (**Figure 3C**). But the activated TEPs did undergo a noticeable change in gene set compared to the activated WTPs (**Figure 3D**). Gene ontology (GO) analysis showed that TEPs showed a significant difference in cell response to stimuli, actin filament, and extracellular vesicles (**Supplementary Figures 2A, B**). The RNA transcript of activated WTPs and TEPs indicated that genes associated with cell adhesion, oncogenes, and immune cell cytokines were upregulated in activated TEPs (**Supplementary Figures 2A, B**).

The gene heat map showed that TEPs highly expressed cell adhesion genes like Itgb2, Tgfbi, Gp5, and Ache (**Figure 3E**). The cell migration-associated skeleton genes, Dnaaf5, Rhog, Parvb, and Tubb5, were significantly enriched in the activated TEPs. The actin-filament genes were upregulated, but those cytoskeleton genes like tubulin and cilium were highly expressed. The highly expressed calcium channel, ATP synthesis, and mitochondrial genes in TEPs may facilitate their potent migration. The vesicle assembly, endocytosis genes, and pro-/oncogenes were upregulated in the activated TEPs. However, the TEP also contains some antitumor genes like Lgals3bp, Ndrg1, Camta3, and Pbrm1. Except these, we also found that TEP expressed Hjv, Psme4, Sbno2, and Zfpm. These genes participate mainly in the biological process like iron homeostasis, DNA damage, osteoblast differentiation, megakaryocyte, and red cell differentiation. The ubiquitination signal pathway genes also were upregulated in TEPs.

Our Gene Set Enrichment Analysis (GSEA) revealed that ATP synthesis, excitatory synapse assembly, postsynapse assembly, and respiratory transport chain were upregulated in activated platelets compared to inactivated wild-type platelets (**Supplementary Figure 3A**). TEPs differed strongly from wild-type platelets in upregulated gene expression associated with the glucose catabolic, microtubule polymerization regulation, vesicle lumen, and abnormal platelet volume (**Supplementary Figure 3B**). The intrinsic pathway of blood coagulation, the assembly of the mitochondrial respiratory chain complex, and the assembly of the motile cilium were strongly expressed in the activated TEPs compared with the TEPs (**Supplementary Figure 3C**). The activated TEPs also exhibited marked upregulation of coagulation, homotypic cell–cell adhesion, actin filament, and vesicle lumen compared with the activated wild-type platelets (**Supplementary Figure 3D**).

## Discussion

The hypercoagulable state in cancer patients is an important reason that may induce cancer-associated thrombus (22). Although many have reported that increasing tissue factors and aberrant coagulant factors contribute to this phenomenon, very little is known about how tumor-educated platelets function in the process. Furthermore, even less is studied on what makes these TEPs different. Here we showed that TEPs are more easily activated and more eagerly form clots when stimulated. Nonetheless, the tumor-derived plasma could more efficiently prevent the platelets from migrating faster and longer. Our mRNA transcript profile further illuminates TEP’s genetic difference, which exhibits a higher expression of genes associated with cell migration and skeleton.

Although the TEPs can migrate potently, they cannot manage that when staying in their original plasma. Normally, the thrombin–antithrombin complex (TAT) is simultaneously produced to inhibit thrombin activity. Lundbech’s study suggests that cancer patients suffer from a hypercoagulable state and simultaneously come with a high anticoagulant ability (23). A higher concentration of mucin and tissue factor in cancer patients would lead to abnormal platelet aggregation, which has also been reported (22). The easily affected state of proteins in plasma may explain why the TEPs cannot migrate faster in WTPL than in their plasma.

Based on our locomotion and RNA-seq results, we estimated that the change in TEPs should mainly happen in the abnormal activation of the coagulation-associated signaling pathway; however, many genes referred to as cell migration and cell skeleton are upregulated instead.

Cancer-associated thrombosis is an urgent problem that needs to be solved. In our study, through the platelet migration assay and RNA-seq profile, we to some extent uncovered the role of tumor-educated platelets in thrombus formation. This example may provide a new insight for understanding and further treating cancer-associated thrombosis. Nonetheless, as the thrombus formation *in vivo* results from coagulation and anti-coagulation, many occult questions still need to be studied.

## Data Availability Statement

The datasets presented in this study can be found in online repositories. The names of the repository/repositories and accession number(s) can be found in the article/**Supplementary Material**.

## Ethics Statement

The studies involving human participants were reviewed and approved by the (B2020-330R) Zhongshan Hospital Research Ethics Committee. The patients/participants provided their written informed consent to participate in this study. The animal study was reviewed and approved by Zhongshan Hospital, Fudan University, Application for Ethical Approval for Research Involving Animals. Written informed consent was obtained from the owners for the participation of their animals in this study. Written informed consent was obtained from the individual(s) for the publication of any potentially identifiable images or data included in this article.

## Author Contributions

YY, ZL, and QS contributed to the conception and design of the study. ZL, FL, and JW organized the database and performed the statistical analysis. ZL wrote the first draft of the manuscript. JW, FL, and YY wrote sections of the manuscript. All authors contributed to the manuscript revision and read and approved the submitted version.

## Funding

Beijing Health Alliance Charitable Foundation supported this work (No. YXKY-WS834B).

## Conflict of Interest

The authors declare that the research was conducted in the absence of any commercial or financial relationships that could be construed as a potential conflict of interest.

## Publisher’s Note

All claims expressed in this article are solely those of the authors and do not necessarily represent those of their affiliated organizations, or those of the publisher, the editors and the reviewers. Any product that may be evaluated in this article, or claim that may be made by its manufacturer, is not guaranteed or endorsed by the publisher.

## Supplementary Material

Supplementary Figure 1 The thromboela-stogram (TEG) and platelet aggregation assay data **(A)** TEG schematic diagram. α: alpha angle (α), the angle between the baseline at initial clot formation and a tangent line that intersects the tracing curve. K, Time from initial clot formation until reaching 20 mm in amplitude. R, Time to initial clot formation (amplitude deviation from baseline). MA, Maximum deviation of tracing to baseline; student t-test; ns, non-significance; **(B)** In TEG assay, cancer patients presented with less reaction time; **(C)** higher coagulation index; **(D)** more significant alpha angle (n=100); **(E)** The K time had no significant difference; **(F)** The maximum amplitude of cancer patients group also showed no noticeable difference; **(G)** In platelet aggregation assay, cancer patients exhibited more significant average aggregation rate; **(H)** also had an enormous maximum aggregation rate; **(I)** when activated by ADP, the average aggregation rate showed no significant difference from the control group; **(J)** The maximum aggregation rate did not increase in cancer patients group; **(K)** The mean platelet volume is more considerable in cancer patients group when activated by arachidonic acid (AA); **(L)** When activated by adenosine diphosphate (ADP), the cancer patients presented with bigger mean platelet volume (n=100); mean ± SEM; ANOVA test; *p < 0.05; **p < 0.01.

Supplementary Figure 2 Gene Ontology of differential expression genes. **(A)** The biological process of GO enrichment. **(B)** The cellular component of GO enrichment. **(C)** The molecular function of GO enrichment. WTP, wild-type platelet; TEP, tumor-educated platelet.

Supplementary Figure 3 Gene Set Enrichment Analysis data (GSEA). **(A)** The genes enriched in pathways associated with ATP synthesis coupled electron transport, excitatory synapse assembly, postsynapse assembly, and respiratory electron transport chain when wild-type platelets were activated; **(B)** Compared to wild-type platelets, the genes of TEPs abundantly enriched in pathways associated with glucose catabolic process, regulation of microtubule polymerization, vesicle lumen, and abnormal platelet volume; **(C)** In activated TEPs the genes enriched mainly in pathways like blood coagulation intrinsic pathway, mitochondrial respiratory chain complex assembly, motile cilium assembly, synaptic vesicle priming; **(D)** When compared to activated wild-type platelets, genes of activated TEPs mainly enriched in coagulation, homotypic cell-cell adhesion, actin filament, and vesicle lumen. WTP, wild-type platelet; TEP, tumor-educated platelet.

Supplementary Video 1 WTP migration in Tyrode’s buffer.

Supplementary Video 2 TEP migration in Tyrode’s buffer.

Supplementary Video 3 WTP migration in WTPL.

Supplementary Video 4 TEP migration in TPL.

Supplementary Video 5 WTP migration in TPL.

Supplementary Video 6 TEP migration in WTPL. WTP, wild-type platelet; TEP, tumor-educated platelet; WTPL, wild-type mice plasma; TPL, tumor mice plasma.
