# Supplementary figures and images for: Tumor-Educated Platelets Facilitate Thrombus Formation Through Migration
Source: Front Oncol. 2022 Feb 24;12:857865. doi: 10.3389/fonc.2022.857865 (PMC8907878; doi:10.3389/fonc.2022.857865)

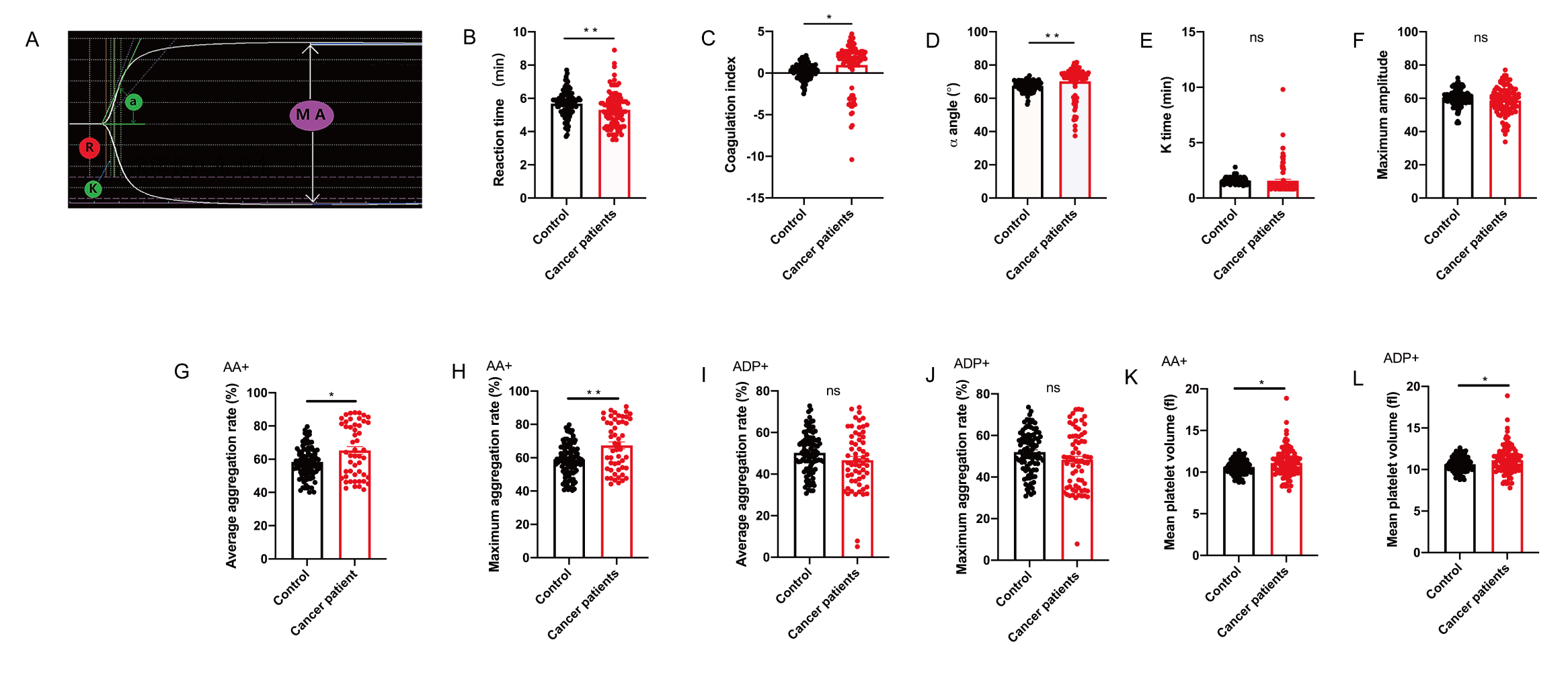

Supplement: Supplementary Figure 1 — The thromboela-stogram (TEG) and platelet aggregation assay data (A) TEG schematic diagram. α: alpha angle (α), the angle between the baseline at initial clot formation and a tangent line that intersects the tracing curve. K, Time from initial clot formation until reaching 20 mm in amplitude. R, Time to initial clot formation (amplitude deviation from baseline). MA, Maximum deviation of tracing to baseline; student t-test; ns, non-significance; (B) In TEG assay, cancer patients presented with less reaction time; (C) higher coagulation index; (D) more significant alpha angle (n=100); (E) The K time had no significant difference; (F) The maximum amplitude of cancer patients group also showed no noticeable difference; (G) In platelet aggregation assay, cancer patients exhibited more significant average aggregation rate; (H) also had an enormous maximum aggregation rate; (I) when activated by ADP, the average aggregation rate showed no significant difference from the control group; (J) The maximum aggregation rate did not increase in cancer patients group; (K) The mean platelet volume is more considerable in cancer patients group when activated by arachidonic acid (AA); (L) When activated by adenosine diphosphate (ADP), the cancer patients presented with bigger mean platelet volume (n=100); mean ± SEM; ANOVA test; *p < 0.05; **p < 0.01. [file Image_1.tif]

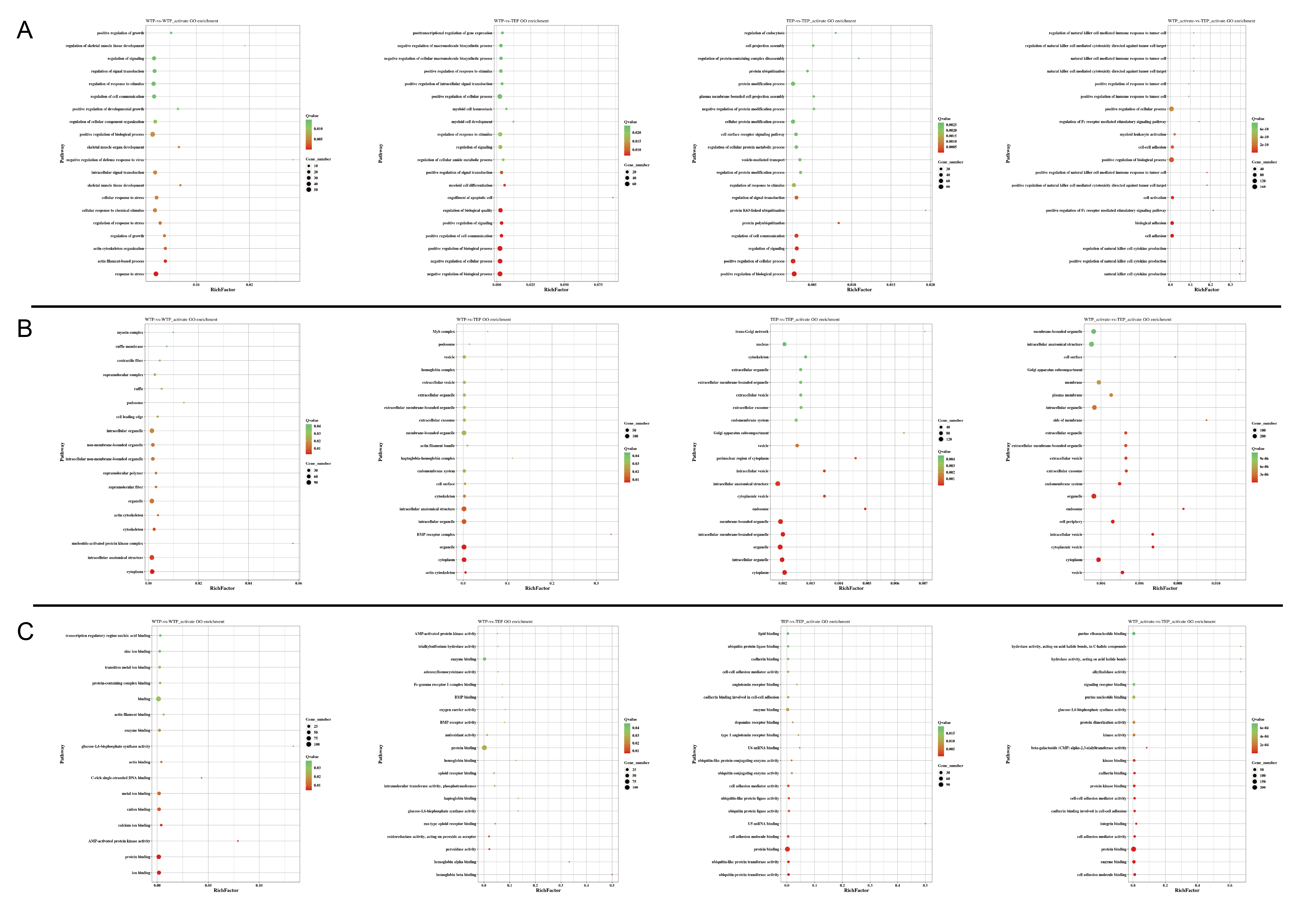

Supplement: Supplementary Figure 2 — Gene Ontology of differential expression genes. (A) The biological process of GO enrichment. (B) The cellular component of GO enrichment. (C) The molecular function of GO enrichment. WTP, wild-type platelet; TEP, tumor-educated platelet. [file Image_2.tif]

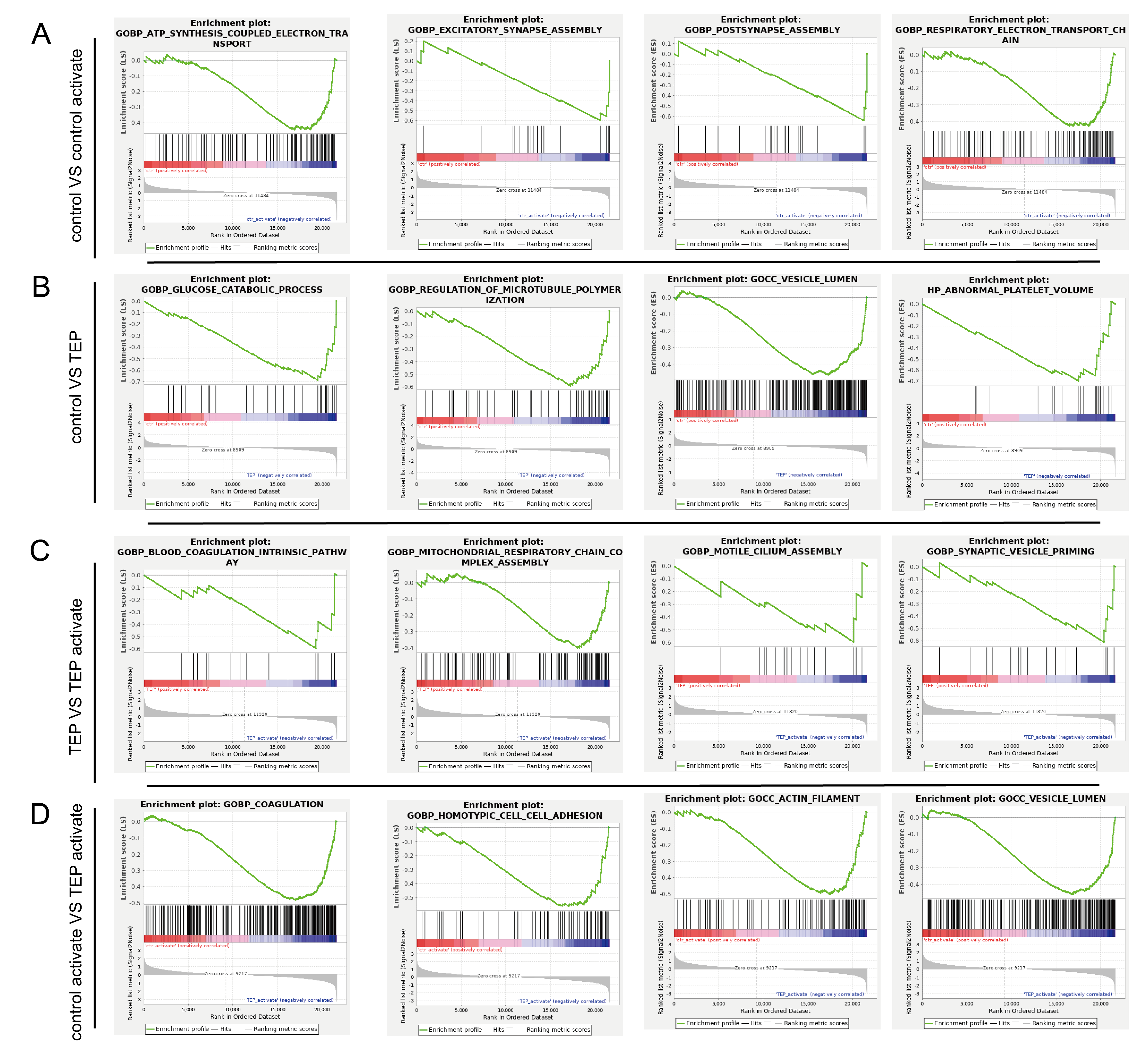

Supplement: Supplementary Figure 3 — Gene Set Enrichment Analysis data (GSEA). (A) The genes enriched in pathways associated with ATP synthesis coupled electron transport, excitatory synapse assembly, postsynapse assembly, and respiratory electron transport chain when wild-type platelets were activated; (B) Compared to wild-type platelets, the genes of TEPs abundantly enriched in pathways associated with glucose catabolic process, regulation of microtubule polymerization, vesicle lumen, and abnormal platelet volume; (C) In activated TEPs the genes enriched mainly in pathways like blood coagulation intrinsic pathway, mitochondrial respiratory chain complex assembly, motile cilium assembly, synaptic vesicle priming; (D) When compared to activated wild-type platelets, genes of activated TEPs mainly enriched in coagulation, homotypic cell-cell adhesion, actin filament, and vesicle lumen. WTP, wild-type platelet; TEP, tumor-educated platelet. [file Image_3.tif]
